# Editorial: Complementary and alternative therapies for sleep disorders: from bench to bedside

**DOI:** 10.3389/fmed.2024.1510996

**Published:** 2024-10-30

**Authors:** Jinhuan Yue, Qinhong Zhang, Guo-qing Zheng, Xiaoqing Zhou, Hao Chi, Hui-Tzu Yang, David M. Zheng, Tiancheng Xu, Brenda Golianu, Guanhu Yang

**Affiliations:** ^1^Vitality University, Hayward, CA, United States; ^2^Heilongjiang University of Chinese Medicine, Harbin, China; ^3^Department of Neurology, First Affiliated Hospital, Zhejiang Chinese Medical University, Hangzhou, China; ^4^Department of Acupuncture, Beijing University of Chinese Medicine Shenzhen Hospital (Longgang), Shenzhen, China; ^5^Clinical Medical College, Southwest Medical University, Luzhou, China; ^6^Faculty of Chinese Medicine, Macau University of Science and Technology, Macau, China; ^7^Key Laboratory of Acupuncture and Medicine Research of Ministry of Education, Nanjing University of Chinese Medicine, Nanjing, China; ^8^Department of Anesthesia, Stanford University School of Medicine, Palo Alto, CA, United States; ^9^Research Department, Swiss Traditional Chinese Medicine (TCM) University, Bad Zurzach, Switzerland; ^10^Department of Specialty Medicine, Ohio University, Athens, OH, United States

**Keywords:** sleep disorder, complementary and alternative therapy, Traditional Chinese Medicine, acupuncture, mechanism, efficacy, safety

Sleep disorders, such as insomnia, obstructive sleep apnea (OSA), and sleep initiation and maintenance disorders (SIMDs), pose a critical public health challenge worldwide, significantly affecting the physical, emotional, and cognitive wellbeing of individuals (1). Although pharmacological treatments remain a common therapeutic option, their long-term efficacy is often compromised by adverse effects and low patient adherence (2, 3). This has led to a growing interest in complementary and alternative therapies (CAT), which provide holistic, non-pharmacological approaches aimed at improving sleep quality while addressing associated comorbidities.

CAT encompasses several interventions, each of which employs distinct mechanisms to enhance sleep health. Among these, acupuncture has emerged as a prominent intervention for both primary insomnia and secondary insomnia associated with comorbidities such as hypertension and cancer. In addition to improving sleep quality, acupuncture has demonstrated benefits in regulating blood pressure, and enhancing cardiovascular health, supporting its inclusion in holistic treatment plans. Similarly, exercise-based interventions, especially when integrated with sleep education programs, offer significant benefits for individuals with OSA. Exercise alleviates the severity of OSA and enhances overall health, making it a viable non-pharmacological alternative to conventional therapies.

Beyond physical therapies, arts-based interventions—including music therapy, Tai Chi, and meditation—offer dual benefits by enhancing sleep quality and addressing mental health conditions such as anxiety and depression. These therapies align with the principles of patient-centered care, providing a comprehensive approach that addresses both psychological and physiological needs. This makes them particularly effective for individuals who are sensitive to medication-related side effects. CAT offers safer, more personalized alternatives that can fill critical gaps in conventional treatments, advancing patient care and improving long-term health outcomes.

This editorial presents nine recent studies conducted by 81 researchers from five countries that highlight the translational potential of CAT from research to clinical application. These studies encompass a spectrum of approaches, from acupuncture and exercise-based interventions to art therapies and the integration of machine learning with Traditional Chinese Medicine (TCM). Taken together, these findings demonstrate how different CAT methods can enhance sleep outcomes across different populations and clinical contexts.

For example, Li et al. employed machine learning algorithms to predict insomnia severity with high accuracy using TCM constitutional classifications. Their study illustrates the intersection of modern technology and traditional medicine, offering personalized insomnia interventions. Similarly, Rodríguez-Aragón et al. examined the impact of Global Postural Re-education on stress and sleep quality in female university lecturers. Their findings suggest that postural correction and body alignment may serve as practical, non-invasive strategies to enhance sleep and reduce stress.

Further evidence supporting exercise-based interventions comes from Fank et al., who examined the effects of combining exercise with sleep education in older adults with OSA. Their results underscore the value of structured physical activity, highlighting its potential to reduce OSA severity and promote better overall health without relying on medication. Acupuncture also plays a central role in the treatment of sleep disorders in patients with complex medical profiles. Zhang et al. conducted a systematic review and meta-analysis, confirming that acupuncture not only improves sleep quality but also reduces blood pressure, offering a dual therapeutic benefit.

Yu et al. investigated the use of weighted blankets as a non-pharmacological tool to enhance sleep. Their review found that weighted blankets, through deep pressure stimulation, were effective in improving sleep quality in individuals with insomnia, autism spectrum disorder, and attention deficit hyperactivity disorder. Huang et al. provided further insight into the global landscape of acupuncture research through a bibliometric analysis. Their findings reveal the increasing adoption of electroacupuncture for the treatment of secondary insomnia and other sleep-related disorders, such as restless legs syndrome, while emphasizing the importance of international collaboration and standardized protocols.

The role of acupuncture in the management of cancer-related insomnia (CRI) was investigated by Chen et al., who conducted a network meta-analysis comparing different acupuncture modalities. They identified auriculotherapy combined with moxibustion as the most effective non-invasive treatment for CRI, underscoring the need for further research on acupuncture as a complementary therapy in cancer care. Sleep disturbances among university students have also garnered attention, particularly in Africa, where Nakie et al. found a high prevalence of poor sleep quality linked to stress, excessive use of electronic devices at bedtime, and chronic illness. These findings call for targeted interventions to address environmental and behavioral contributors to sleep disorders.

Finally, Luo et al. explored the use of art therapies, such as music therapy, Tai Chi, and meditation, for treating SIMDs. Their review found that these therapies not only improved sleep quality but also alleviated underlying mental health issues, such as anxiety and depression, supporting their adoption as holistic treatment options in clinical practice.

The contributions within this editorial collectively highlight the expanding evidence base for CAT as a viable intervention for sleep disorders. These approaches reflect the innovative, multidisciplinary strategies being developed to address sleep disturbances in diverse populations. As research in this field continues to evolve, the integration of CAT into clinical practice offers the potential to improve patient care by providing safer, more personalized treatments for sleep disorders and promoting overall health outcomes.
